# Implementation and Evaluation of a Collaborative, Pharmacy-Based Hepatitis C and HIV Screening Program

**DOI:** 10.5888/pcd19.220129

**Published:** 2022-12-08

**Authors:** Donald G. Klepser, Michael E. Klepser, Philip J. Peters, Karen W. Hoover, Paul J. Weidle

**Affiliations:** 1 College of Pharmacy, University of Nebraska Medical Center, Omaha, Nebraska; 2 College of Pharmacy, Ferris State University, Kalamazoo, Michigan; 3 Division of HIV/AIDS Prevention, National Center for HIV, Viral Hepatitis, STD, and TB Prevention, Centers for Disease Control and Prevention, Atlanta, Georgia

## Abstract

**Introduction:**

Pharmacy-based HIV and hepatitis C virus (HCV) screening services developed in conjunction with state and local health departments can improve public health through increased access to testing and a linkage-to-care strategy. The objective of this study was to evaluate the impact of implementing HIV and HCV screening in community pharmacies.

**Methods:**

This prospective, multicenter implementation project was conducted from July 2015 through August 2018. Sixty-one pharmacies participated in 3 US regions. We assessed the effectiveness of point-of-care testing, counseling, and disease education for populations at increased risk for HIV and HCV infection through screening programs offered in community pharmacies. Pharmacy customers were offered screening with point-of-care HIV and/or HCV tests. Reactive test results were reported to state or local health departments for disease surveillance.

**Results:**

A total of 1,164 patients were screened for HIV, HCV, or both at the 61 participating pharmacies; the average number of patients screened per pharmacy was 19. Pharmacists conducted 1,479 HIV or HCV tests among the 1,164 patients. Five of 612 (0.8%) HIV tests yielded a reactive result, and 181 of 867 (20.9%) of HCV tests yielded a reactive result.

**Conclusion:**

Patients at increased risk of HIV or HCV can benefit from screening for infection at community pharmacies. Ease of accessibility to testing coupled with a strategy for linkage to care designed for the local community can improve patient care and improve the course of treatment for HIV and HCV.

**Summary**

**What is already known on this topic?**

One strategy to reduce the incidence of HIV and hepatitis C virus (HCV) is to increase the number of screenings of people at increased risk of infection. Pharmacies are often identified as the most accessible entry point for patients into the US health system.

**What is added by this report?**

Community pharmacy–based HIV and HCV screening programs created in partnership with state public health departments can be implemented in both urban and rural communities.

**What are the implications for public health practice?**

Community pharmacy and public health partnerships can improve access to HIV and HCV screening and help link people previously unaware of their status for treatment if needed.

## Introduction

HIV and hepatitis C virus (HCV) have affected millions of people in the US (1). Although HIV and HCV infections have been historically associated with poor prognoses, advances in treatment have significantly decreased the illness and death associated with these viruses (2,3). Currently, the primary factor limiting care for people infected with HIV or HCV is the recognition of infection and establishment of care.

In 2018, 14% of people with HIV infection in the US were unaware of their diagnosis (4). Of those who were diagnosed with HIV infection in 2018, roughly 23% did not receive treatment (4). Similarly, approximately half of people with chronic HCV infection in the US in 2018 were unaware of their diagnosis. Many people who are aware of their HCV infection never receive treatment because they are either lost to follow-up or do not meet criteria for treatment. That many people with HIV or chronic HCV infection are not receiving treatment is a public health concern (2,3). One shortcoming of the US health care system is the ability to link to appropriate care a person who receives a positive test result for HIV or HCV. When the establishment of care is left to the patient or managed passively by health care providers, patients may become overwhelmed. As a result, patients often become discouraged and do not receive follow-up care. The lack of follow-up care often results from the confusion created by attempting to navigate a complicated health care system and unclear instructions on how to access health care providers who could manage HIV or HCV infection.

One way to decrease the prevalence of HIV and HCV infection is to increase screening rates among people at increased risk for these infections and improve treatment rates. The National HIV/AIDS Strategy for the United States and the National Viral Hepatitis Action Plan have stressed both increased access to screening and improved linkage to care (2,3). A care strategy that has been proposed is a “warm” handoff from the health care provider who conducts the screening to the health care provider who initiates and manages treatment. This strategy aims to make entry into care personal and efficient, which can reduce the amount of time to treatment. If more people with HIV and chronic HCV infection establish care and receive treatment, not only will their health outcomes improve but further transmission of HIV and HCV will slow.

Pharmacies are often identified as the most accessible entry point into the US health care system. There are roughly 62,000 retail pharmacies and more than 180,000 pharmacists practicing in community settings in the US. Furthermore, an estimated 91% of all people in the US live within 5 miles of a community pharmacy (5). Given their accessibility, community pharmacies have been proposed as potential screening sites for HIV and HCV. Studies of pharmacy-delivered HIV or HCV screening services have focused on either HIV or HCV screening, have described the use of nonpharmacy staff members for the screenings, or have described screenings held as special events (6–11).

Pharmacy-based HIV and HCV screening services developed in conjunction with state and local health departments can improve public health by increasing access to testing and offering a clear linkage-to-care strategy designed for the local community. The objective of this study was to evaluate the impact of implementing HIV and HCV screening services in a community pharmacy setting. This was a proof-of-concept project to demonstrate that testing services could be effectively developed and implemented in pharmacies and provide justification for allowing us to address effect and linkage to care in a future project.

## Methods

This implementation study examined the impact of point-of-care HIV and HCV testing, counseling, and disease education on populations at increased risk for HIV and HCV infection through screening programs offered in community pharmacies. Points of access to screening were provided in both rural and urban settings at pharmacies in 4 states: Georgia, Michigan, Ohio, and West Virginia. State and local health departments were engaged early in the process to ensure that patients who received a reactive test result could be connected to care for confirmatory testing and follow-up care via existing networks and resources; the health departments worked with pharmacies to develop and implement patient education and reporting programs and linkage-to-care procedures. The protocol and procedures were reviewed by the University of Nebraska Medical Center institutional review board and deemed exempt.

This prospective, multicenter implementation project was conducted from July 2015 through August 2018. Sixty-one pharmacies in 3 regions participated in the study. Patient recruiting and screening began with pharmacies in Detroit, Michigan. Sites were selected according to accessibility to populations at risk of HIV and HCV infection, walk-in patient volumes, and having a pharmacist at the site with the training necessary to assess and test patients. The study expanded to 2 additional regions: 1) Huntington/Charleston, West Virginia, along with Ohio communities neighboring the West Virginia border, and 2) Atlanta, Georgia. Pharmacies were provided tests, supplies, and training as part of the project but were not paid or reimbursed for tests conducted. For the purposes of the project, pharmacists understood the importance of HIV and HCV screening and contributed their time as a public service.

### Study population

Testing was focused on populations in greatest need of HIV and HCV screening. For the purposes of the study, these populations included patients meeting at least one of the criteria outlined by the study team (Table 1). Patients were ineligible to participate if they were aged 18 years or younger, were unable or unwilling to provide informed consent, or had previously been diagnosed with HIV or HCV infection and were receiving medical treatment for these conditions.

**Table 1:** Characteristics Used to Identify Patients at Increased Risk of HIV or Hepatitis C Virus

Type of infection	Risk
HIV	Any man who reports a male sex partner within the past 12 months
	Any person who reports injecting drugs
	Any heterosexual man or woman who reports either of the following: 1) multiple partners and inconsistent condom use within the past 12 months, or 2) history of a sexually transmitted infection (syphilis or gonorrhea) within the past 12 months
Hepatitis C virus	Any person who reports injecting drugs
	Any person who has HIV
	Any person who has received a piercing or tattoo in an unclean environment using unsterile equipment
	Any person who received a blood transfusion or organ transplant before 1992
	Any person who has ever been in prison
	Any person born between 1945 and 1965, the age group with the highest incidence of hepatitis C infection

Patients were recruited for the study through passive and active recruitment strategies in the participating pharmacies. The passive recruitment approach to routine testing consisted of educational materials describing when and for whom HIV and HCV screenings are recommended and the availability of testing at the pharmacy. For HCV testing, active recruitment involved patients born from 1945 through 1965 (3). Pharmacists also partnered with local public health departments to make patients aware of the screenings and to provide off-site screening events.

Consent to test was obtained from all eligible patients before further participation in the study. Once eligible patients were identified and consent was obtained, contact information, demographic data, and past medical history were collected. A risk assessment for HIV and HCV was completed, and a brief physical assessment including a patient interview and collection of vital signs was performed.

Patients were screened with a US Food and Drug Administration (FDA)–approved, Clinical Laboratory Improvement Amendments (CLIA)–waived rapid HIV test (OraQuick HIV test kit, Orasure Technologies) and FDA-approved, CLIA-waived rapid HCV test (OraQuick HCV test kit, Orasure Technologies). Before conducting the test, all patients were asked to review and sign a document outlining the value and limitations of HIV or HCV screening and what steps were to be taken in the event of reactive test results.

While tests were being processed, all patients received education on HIV and/or HCV. This education consisted of standard pretest and posttest information on 1) HIV and HCV testing (including the concept of a window period — the earliest phase of infection, when a person may have been infected but too early for the test to be reactive), 2) HIV and HCV infection, and 3) risk-appropriate follow-up testing recommendations in accordance with state health department guidelines. Additional risk-reduction counseling was provided when the patient requested it. In addition, patients with a reactive rapid HIV or HCV test result were counseled in accordance with local and federal guidelines.

Upon receipt of test results, the patient was counseled by the pharmacist on the meaning of the findings and provided a hard copy of the results. Additionally, a copy of the test results was faxed to the patient’s primary care provider if one was identified. Patients with a reactive test result were instructed to make an appointment with a physician with experience in the management of patients with HIV or HCV infection. The local health department was contacted to provide partner services for those with reactive HIV tests and linkage to follow-up care. If the patient did not identify a preferred health care provider, the pharmacist offered to help the patient make an appointment with a physician who had been pre-identified as part of the study and was accepting new patients.

State and local health departments were included in research planning calls. Although the testing criteria and in-pharmacy procedures were consistent across sites, reporting and referral criteria were developed in collaboration with each state and local health department. The intent was to work within the existing public health structure in each community. Reactive test results were reported to the relevant state health department for disease surveillance.

### Pharmacist training

Before participating in the screening program, all pharmacists completed an Accreditation Council for Pharmacy Education–accredited 20-hour certificate program on the use and interpretation of point-of-care tests. The training includes modules focused on counseling patients who receive a reactive test result for HIV or HCV.

### Data analysis

We used descriptive statistics to describe the demographic characteristics of patients who were screened, the risk factors of patients screened, the prevalence of previous screening among patients, and the screening results by location. We used SAS OnDemand for Academics (SAS Institute, Inc) for all analyses.

## Results

A total of 1,164 patients were screened for HIV, HCV, or both at the 61 participating pharmacies (Table 2); the average number of patients screened per pharmacy was 19. Among the 612 patients screened for HIV, the mean age was 38.5 years, 307 (50.2%) were female, and 166 (27.1%) reported that they had been previously screened for HIV. Among the 867 patients screened for HCV, the mean age was 46.5 years and 55.7% were female. Of that population, 264 (30.4%) reported that they had been previously screened for HCV.

**Table 2:** Demographic Characteristics of People Screened in a Study Designed to Evaluate the Impact of Implementing HIV and HCV Screening in Community Pharmacies^a^

Characteristic	Total screened (N = 1,164)^b^	Screened for HIV (n = 612)	Screened for HCV (n = 867)
Age, mean (SD), y	44.9 (16.6)	38.5 (13.9)	46.5 (16.7)
Sex at birth, no. (%)			
Female	636 (54.6)	307 (50.2)	483 (55.7)
Male	528 (45.4)	305 (49.8)	384 (44.3)
Previously tested, no. (%)	430 (36.9)^c^	166 (27.1)^d^	264 (30.4)^e^

Abbreviation: HCV, hepatitis C virus.

a The study was conducted from July 2015 through August 2018. Sixty-one pharmacies participated in 3 US locations: Atlanta, Georgia; Detroit, Michigan; and Huntington/Charleston, West Virginia, along with Ohio communities neighboring the West Virginia border.

b Some people were screened for both HIV and HCV.

c Previously tested for either HIV or HCV.

d Previously tested for HIV.

e Previously tested for HCV.

Pharmacists conducted 1,479 tests among the 1,164 patients (Table 3). Five of 612 (0.8%) HIV tests yielded a reactive result, and 181 of 867 (20.9%) of HCV tests yielded a reactive result. Although the percentage of reactive HIV tests was consistently low (<2%) across all locations, the percentage of reactive HCV tests was higher, ranging from approximately 3.5% in Michigan and Georgia to 30.7% in West Virginia. All patients with a reactive test result were given educational materials and referred for confirmatory testing, and all reactive test results were reported to the appropriate state or local health departments. Of patients with a nonreactive test result, 59 received a recommendation to be retested for HIV because of a possible HIV exposure in the previous 3 months, and 13 received a recommendation to be retested for HCV because of a possible HCV exposure in the previous 6 months.

**Table 3:** HIV and HCV Testing and Results, by Location, in a Study Designed to Evaluate the Impact of Implementing HIV and HCV Screening in Community Pharmacies^a^

Location	Pharmacies	HIV tests	Reactive HIV tests	HCV tests	Reactive HCV tests
Michigan, no.	29	104	0	175	6
West Virginia and Ohio, no.	28	331	2	553	170
Georgia, no.	4	177	3	139	5
All, no. (%)	61 (100)	612 (100)	5 (0.8)^b^	867 (100)	181 (20.9)^c^

Abbreviation: HCV, hepatitis C virus.

a Study was conducted from July 2015 through August 2018. Sixty-one pharmacies participated in 3 US locations: Atlanta, Georgia; Detroit, Michigan; and Huntington/Charleston, West Virginia, along with Ohio communities neighboring the West Virginia border.

b Denominator is number of HIV tests (5 of 612).

c Denominator is number of HCV tests (181 of 867).

## Discussion

Our results indicate that pharmacists were able to successfully implement a targeted HIV and HCV screening program in their community. The expanded access to screening provided by the participating pharmacies resulted in 186 reactive tests among previously undiagnosed patients. Those 186 patients were educated about their disease and were referred for confirmatory testing. Moreover, all reactive test results were reported to the appropriate state or local health department so that patients could be monitored and receive follow-up care as appropriate.

Among the participants in our study, 20.9% received a positive test result for HCV infection, which is significantly higher than the 1.7% reported prevalence of HCV infection in the US (12). This high rate of reactive tests is likely due to the focused nature of the screening. In particular, the West Virginia locations participating in the study focused on people who inject drugs, and among this population, the prevalence of HCV infection is higher (12%–20%) than in the general US population. The reactive test rate for HIV infection in our study was less than 1%, which is consistent with the overall prevalence of HIV in the US. Other studies that have examined pharmacy-based HIV and HCV screening programs have reported test positivity rates ranging from 0.8% to 1.6% for HIV and 1.2% to 8.0% for HCV screening (6–8,11). Rates of positivity vary among studies because of the structure of screening programs and the local variability in the prevalence of infection. Regardless, all studies indicated that pharmacy-based screening for HIV and HCV was an effective means for identifying people in need of follow-up care.

Overall, each pharmacy tested on average 19 patients during more than 2 years of data collection. Although some pharmacies screened more patients than others, it is apparent that barriers remain to making the pharmacy a usual setting for testing. The 3 largest barriers to widespread testing are likely pharmacist comfort, patient awareness of the availability of the test, and pharmacy workflow. The typical workflow in a pharmacy is focused on filling prescriptions; a patient requesting a test is an unexpected workflow disruption. All pharmacists participating in the study were trained in counseling and educating patients about HIV and HCV, but some were likely more comfortable than others with screening and counseling. Anecdotally, several pharmacists expressed reservations about having to tell a patient that they had a reactive test result. Their concerns revolved around their own emotions and the emotions of patients. Many of these concerns were alleviated by study investigators and pharmacy managers reiterating to pharmacists that providing test results should be viewed as helping patients obtain needed care rather than as conveying difficult news. We observed that many patients who sought testing for HIV did so because they previously received a reactive test result and were typically not surprised by another reactive test result. Moreover, although the tests themselves are simple to use, both involve finger sticks. Some pharmacists may not be comfortable with any risk of exposure to blood and may be reluctant to perform HIV or HCV screenings. In this study, the decision to provide testing was made at the corporate level of the pharmacy and not by the individual pharmacist.

Other barriers to widespread screening in pharmacies are likely related to creating new models of care in the pharmacy, prioritization of tasks, and unclear policies and procedures for reimbursement. In many pharmacies, staff performance is evaluated based on prescription fill metrics. Point-of-care testing services disrupt the filling process and decrease fill counts. Some pharmacies have tried to develop activity conversions to not penalize staff. One study showed that, of pharmacies offering HIV screening services, pharmacy staff time could be divided into 3 categories: 1) pretest time, which included counseling, consent, and specimen collection, 2) time waiting for test results, and 3) posttest time, which involved sharing test results with patients, providing risk-reduction information to patients with nonreactive test results, or arranging to obtain confirmatory tests for reactive test results (6). That study examined the impact of a pharmacy-based HIV screening program using the OraQuick HIV testing kit (OraSure Technologies) on workflow. It found that pretest time, waiting time, and posttest time were on average 4 minutes, 23 minutes, and 3 minutes, respectively. During the waiting time, staff were not engaged with the patient and were able to return to other activities. Mean posttest times were significantly different between patients with reactive test results (14 minutes) and patients with nonreactive test results (4 minutes). Overall, the study found that the average hands-on time required by pharmacy staff was approximately 7 minutes (6). This amount of time is similar to the amount of time needed to offer other pharmacy-based clinical services such as influenza screening and acute pharyngitis screening (10,13,14).

One observation made in our study is related to reporting test results and reinforces the need to develop testing programs in conjunction with public health departments. Some jurisdictions instructed us to report all tests that were performed. Other jurisdictions required only confirmed tests to be reported. One state requested HCV and HIV test results to be submitted by 2 different methods. The HCV test results were entered into an electronic portal, whereas the HIV test results had to be faxed. This lack of standardization of reporting even within the same state highlights the need for pharmacies to work closely with public health departments when designing screening services.

In 2019, the Centers for Disease Control and Prevention updated the status of HIV testing and viral suppression in the US (15). The report indicated that the percentage of people living with HIV who were diagnosed increased from 83% in 2013 to 86% in 2018. Despite this improvement, the report suggested that the nation will fall short of the goal of reducing new HIV infections by 90% by 2030 unless new strategies are used to screen and manage patients. Our data demonstrate that community pharmacy screenings can play a role in diagnosing HIV and HCV infections to help achieve public health goals.

### Limitations

One limitation to this study is that we were unable to follow patients after they were screened in the pharmacy. Therefore, we do not know how many received confirmatory testing or received treatment. Another limitation of the study is the location of pharmacies selected to participate. Although we selected communities with a high prevalence of HIV or HCV, the pharmacies themselves may not have been in the neighborhoods or areas with a high prevalence, and although the selected pharmacies may have served a medically underserved population, they may not have been conveniently located enough to drive high levels of screening. In addition, we did not conduct an economic analysis; the economics of providing HIV and HCV screening can affect pharmacy adoption and demonstrate the value of an intervention that identifies previously undiagnosed infections. Economic analyses are needed to assess the cost-effectiveness of community pharmacy–delivered screening programs and determine the level of investment that would be required to sustain the model. Finally, we recognize that capturing information on pharmacists’ concerns and perceptions of HIV and HCV testing would have been valuable. Because these data were not systematically captured, we could report only anecdotal findings.

### Conclusion

The collaborative participation and involvement of pharmacists, physicians, and public health officials were key to our study’s success and would be similarly important to the implementation of HIV and HCV screening services outside of a study. Any community pharmacy interested in developing similar services would be well advised to engage state or local public health agencies and physicians early in the process. Likewise, state and local health departments should consider engaging community pharmacies in efforts to expand HIV and HCV screening and linkage to care.

Our study demonstrated that community pharmacies can serve as an important public health partner in the screening of people at increased risk for HIV and HCV infection. Ease of accessibility to testing coupled with a strategy for linkage to care designed for the local community has the potential to improve patient care and significantly affect the course of HIV and HCV infections.
